# A Method for the Experimental Determination of Magnetic Permeability at a Stand for Non-Contact Determination of Temperature Characteristics of Electrical and Thermal Properties of Conductive Materials

**DOI:** 10.3390/ma19102042

**Published:** 2026-05-13

**Authors:** Jerzy Zgraja

**Affiliations:** Institute of Applied Computer Science, Lodz University of Technology, 90-537 Lodz, Poland; jzgraja@p.lodz.pl

**Keywords:** induction heating, material properties, temperature characteristics, magnetization characteristics

## Abstract

The article concerns the issue of simultaneous determination of material parameters of electrical conductors. In reference to previous work on a non-contact measurement station enabling the simultaneous determination of electrical and thermal properties of conductive materials, the concept of its extension to include magnetic measurements was analyzed by adding a module for determining the magnetization characteristics of ferromagnetic conductors, integrated with the station. The accuracy of determining the magnetization characteristics of a flat sample closing a magnetic circuit based on a U-shaped ferrite core with using a triangular excitation signal with a frequency of several kilohertz was analyzed through simulation and initially verified experimentally. Based on the measurement systems used, relationships enabling estimation of the magnetization characteristics were developed, and the influence of the geometric parameters of the magnetic circuit and the excitation signal parameters on the magnitude of the measurement error was presented.

## 1. Introduction

Reliable information concerning material properties is an essential element of the correct implementation of many industrial processes. This also applies to heating processes, which additionally require knowledge of their variation with temperature *T*, i.e., temperature characteristics. For electrically conductive materials, especially metals, induction heating [1] is currently the primary heating method, being used in heat treatment and thermal forming. In this effective and flexible method, the influence of material properties on the obtained heating effect is particularly significant and simultaneously concerns several completely different parameters. These are, primarily, the temperature characteristics of resistivity *ρ*(*T*), specific heat *c*(*T*), and specific thermal conductivity *k*(*T*), and, for ferromagnetic materials, the magnetization characteristics *B*(*H*), i.e., the dependence of magnetic induction B on the magnetic field strength *H*, which can be translated into the dependence of magnetic permeability *μ*(*H*) on the magnetic field strength.

Information concerning the properties or temperature characteristics of these material properties can be obtained in various ways. The simplest method is to use information contained in material catalogs, if available. On the other hand, a professional approach based on the measurement of characteristics, and in particular parameters as diverse as electrical and thermal parameters, often encounters significant hardware barriers and usually requires expensive and time-consuming measurements in external measurement laboratories. This indicates a lack of user-friendly devices that allow for the rapid (preferably “in situ”) and most accurate determination of temperature characteristics, ideally simultaneously determining several material properties. The literature presents attempts [2,3,4,5,6,7,8,9,10,11,12] to accomplish this task, indicating a real need. These attempts mainly concern thermal properties of materials, i.e., thermal diffusivity and heat capacity (specific heat). In some of them [2,3], the influence of temperature on the determined parameters is completely ignored or strongly marginalized [4] or only dielectric materials are considered [3]. When using contact heating methods, the accuracy of the measurements is additionally limited [5] by the influence of the thermal resistance of the connections, which is difficult to eliminate. In recent years, promising literature [6,7] has emerged on measurement techniques that allow for the simultaneous determination of temperature characteristics of specific heat and thermal conductivity of metals with an accuracy of several percent. However, these techniques rely on the use of a resistive, contact heat source, which makes them less useful at higher temperatures encountered in metalworking processes. The described techniques for the simultaneous determination of several material parameters are based on solving the inverse heat conduction problem, i.e., searching for such values of material parameters that, when solving the thermal conductivity equation, give temperature courses consistent with the measured courses of the temperature response of the tested material to the applied forcing. A similar approach to studying the temperature response of the tested sample, but using non-contact induction heating, was presented in [8,9]. Conclusions from the conducted work indicate that the simultaneous determination of several material parameters based on temperature measurements and optimization techniques is subject to a significant risk of error resulting from the similar influence of the determined parameters on the objective function, which can lead to ambiguous solutions. This risk increases as the permissible range of changes in the decision variables widens (i.e., values of the searched parameters). This leads to the need for either expert knowledge or a preliminary narrowing, based on measurements, of the permissible range of changes in the values of the searched parameters. The concept of such a measuring station enabling the simultaneous determination of temperature characteristics of several different material parameters in one automatically controlled measurement cycle was analyzed in [11,12]. Assuming that the density value *ρ*_m_ of the tested material is known, the station allows for simultaneous determination (in one measurement cycle) of temperature characteristics of thermal capacity *χ*(*T*) = *c*(*T*)·*ρ*_m_ and thermal diffusivity *a*(*T*) = *k*(*T*)/*χ*(*T*), as well as the resistivity *ρ*(*T*) of electrical conductors (which also allows determining the specific heat *c*(*T*) and *k*(*T*) specific heat conductivity).

This article analyzes the possibility of expanding the above-mentioned stand [12] with a module for determining the magnetization characteristics *B*(*H*) integrated with both hardware and software. This will expand the measurement capabilities not only to include the *B*(*H*) characteristic but also enable the simultaneous measurement of temperature characteristics of electrical and thermal parameters for ferromagnetic materials, as described above. The integration of the *B*(*H*) measurement module with the entire stand expands the measurement possibilities but also introduces a number of limitations regarding the possible techniques for measuring magnetization characteristics, which is the subject of this article. In order to better understand the importance and, at the same time, the limitations resulting from incorporating the *B*(*H*) measurement module into the system being built for measuring the temperature characteristics of electrical and thermal parameters of conductive materials, this article also briefly presents the operating principles of this system.

It should be noted that the presented measurement system may, depending on the required accuracy in determining the material parameters, be either be treated as a final measurement station, or can be further expanded with additional procedures based on the optimization process and the solution of the inverse heat conduction problem to improve its accuracy; this an element of the work is currently underway.

## 2. A Measuring Station for the Simultaneous Determination of Electrical and Thermal Properties

The developed measurement stand utilizes a resonant generator, which is typical for industrial induction heating applications, as a source of excitation signals. Modern industrial induction heating stations typically comprise a complex system containing both an electronic power source and sophisticated automation systems that control drives and cooling systems, among other things. Basing the concept of a stand for measuring material properties on a structure similar to that of a typical induction heating station creates the possibility of future expansion of such industrial induction heating systems with an additional block for determining the properties of charge materials.

**Figure 1:**
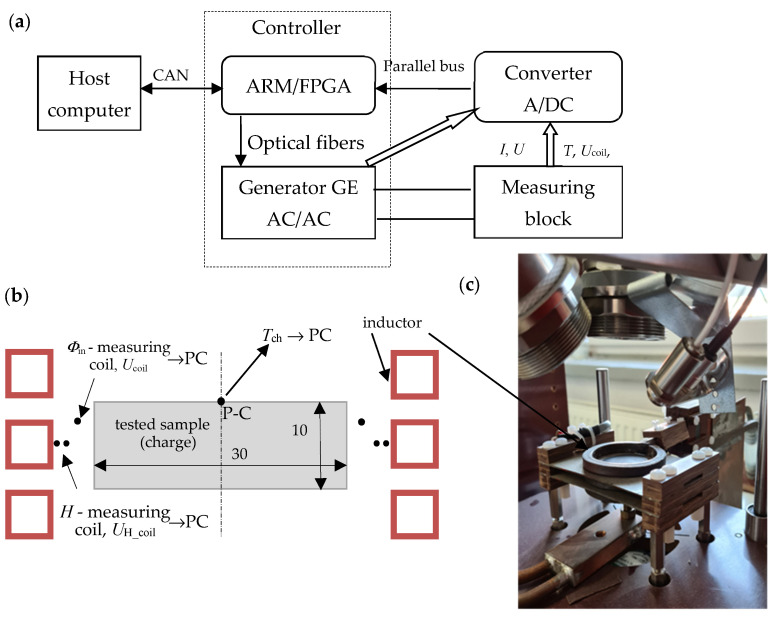
Stand for measuring electrical and thermal material properties: (**a**) block diagram; (**b**) illustrative view of the measurement sample location; (**c**) actual view of the measurement block.

Figure 1a shows a block diagram of the stand for the simultaneous measurement of temperature characteristics of electrical and thermal properties of conductive materials; Figure 1b shows an illustrative view of the location of the measuring sample; and Figure 1c shows a photo of the constructed measuring block.

**Figure 2:**
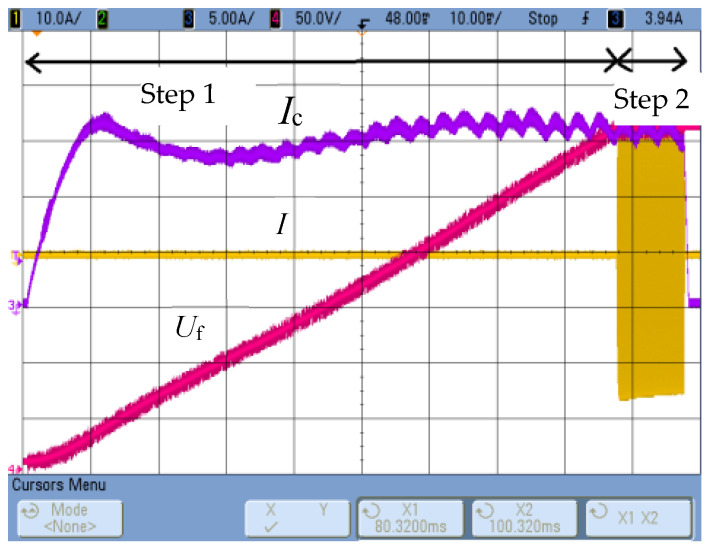
The waveform of the inverter source voltage *U*_f_, chopper choke current *I*_c_ and inverter current *I*.

The measurement of thermal properties in the discussed system involves determining the thermal diffusivity and thermal capacity of the tested material. A cylindrical sample with a radius of *R* = 15 mm and a thickness of *g* = 10 mm (Figure 1b) is tested, placed in a three-turn cylindrical inductor. Thermal diffusivity is measured by modifying the Flash pulse method [13] used in professional laser thermal diffusivity measurement devices. In the original Flash method [13], the dynamics of the temperature response on the front surface of a cylindrical sample caused by incident electromagnetic pulse excitation (close to a Dirac pulse) on its opposite side are studied. Thermal diffusivity is determined based on the half-response time *t*_1/2_, i.e., the time after which half the maximum temperature value is obtained. In the constructed system, both the configuration of the measurement system and the method of implementing the excitation were modified, which eliminated the possibility of using the computational relations given in the literature [13,14]. The excitation was assumed to be an excitation current pulse, as close to rectangular as possible (Figure 1b), generating a power pulse in the area close to the lateral surface of the cylindrical sample. The time course of the temperature response *T*_ch_(*t*) was recorded at the central control point P-C (Figure 1b) of the front surface. The energy of the generated pulse depends on both the value and frequency of the excitation current, the resistivity and magnetic permeability of the tested material, and the excitation duration, which should be as short as possible. Considering the possible wide range of resistivity changes in the tested materials, and the need for the highest possible measurable temperature response, the excitation current frequency was set [11] at approximately *f* = 40 kHz, and the pulse duration at Δ*t* = 10 ms. The initial effective value of the excitation current was set at *I* = 1550 A, although this value (depending on the actual resistivity and magnetic permeability values of the tested sample material) can be corrected as a result of the initial test. Thanks to the developed control algorithm [11], it is possible to achieve step changes in the inverter source current close to a rectangular pulse; see Figure 2.

For a wide range of variations of the tested electrical and thermal material parameters, a relationship was established [11] that allows for the determination of the thermal diffusivity of the non-magnetic sample material based on the time *t*_1/2_ of the temperature response at point P-C. In [12], this relationship was corrected in a way that makes it possible, with an error not exceeding 5%, to determine the thermal diffusivity of both magnetic and non-magnetic materials:

(1) $$a=0.104\frac{{RR}_{n}^{2}}{t_{1/2}-0.75\cdot ∆t}$$

where: *RR*_n_ = *R* − *δ*/4, and *δ*—is the value of the penetration depth of the electromagnetic wave into the tested material at the applied frequency of the exciting current.

The mathematical relationship for the penetration depth *δ* of an electromagnetic wave is defined [15] by the following formula:

(2) $$\delta =\sqrt{\frac{2\cdot \rho }{\omega \cdot \mu }}$$

where: *ω* = 2·*π*·*f*—current pulsation.

Using Equation (1) requires knowledge of the resistivity value and, for magnetic materials, also the magnetic permeability. The resistivity value is determined on this test stand in the same measurement cycle, and therefore for the same base temperature as the diffusivity. The radial temperature difference in the sample induced by the measurement pulse is a maximum of a few degrees, which has no practical effect on the resistivity value. In the case of magnetic materials, the magnetic permeability value of the tested material needs to already be known at the stage of determining thermal properties, which can pose a significant problem. This requires either prior knowledge of the magnetization characteristics of the tested material or its prior measurement. It was assumed that the measurement station would be equipped with a system for measuring permeability *μ*(*H*), and that this measurement would be performed in a separate, earlier measurement cycle for the ambient temperature, which will be discussed in this article. It should be noted that the diffusivity measurement is performed for relatively high values of the inductor current, which causes magnetic field strengths of the order of hundreds of kA/m, and consequently low values of relative magnetic permeability of the tested sample material. As presented in [12], in this case, the magnetic nonlinearity into the load will also be of little quantitative significance if the value of relative magnetic permeability is assumed for the effective value of the field strength *H* in the inductor-load gap at half the load height. This value of the magnetic field strength is measured by the measuring coil, as shown in Figure 1b.

The heat capacity *χ* of the tested sample material can be determined based on the time derivative of its temperature response to a step power excitation, according to the following relationship:

(3) $$\chi =\frac{P_{ch}}{V_{ch}\frac{dT_{ch}}{dt}}$$

where: *P*_ch_—power generated in the sample, *V*_ch_—sample volume, and *T*_ch_—sample temperature.

The above relationship is valid provided that the power dissipation density and temperature are uniformly distributed in the sample. For the system shown in Figure 1 and the implemented step excitation of the inductor current, the above conditions are clearly not met. As shown in [11,12], this relationship can be used, however, if it concerns the maximum value of the temperature derivative at the control point P-C. However, the presented method for determining thermal storage capacity poses the problem of correctly determining the value of power *P*_ch_ dissipated in the charge (sample), or essentially the power involved in increasing the enthalpy of the sample material. With a high value of power delivered to the sample, it is possible to neglect energy losses to the environment and assume that all the energy released in the sample goes to increasing its enthalpy. In the presented system, this power is determined as the difference between the power delivered from the source *P*_g_ and the power losses in the inductor *P*_in_:

(4) $$P_{ch}=P_{g}-P_{in}$$

The source power *P*_g_, frequency, and exciter current are measured on the test stand, and the *P*_in_(*I*, *f*) relationship is determined (constant shape and dimensions of the sample) during the test stand calibration. For non-magnetic materials, the dependence *P*_in_(*I*, *f*) determined in this way is practically independent of the resistivity of the sample material, whereas for magnetic materials [12] the influence of the electric-magnetic parameters of the charge (change in the current density distribution in the inductor caused by the change in the magnetic parameters of the sample) cannot be completely neglected. The power losses in the inductor increase with the increase in the permeability of the test sample material, especially at higher values of the charge resistivity, which can be approximated [12] by the functional dependence:

(5) $$P_{in}=P_{in}(\mu_{r}=1)\cdot \mu_{r}^{0.06+0.0035\cdot ln(\rho )}$$

**Figure 3:**
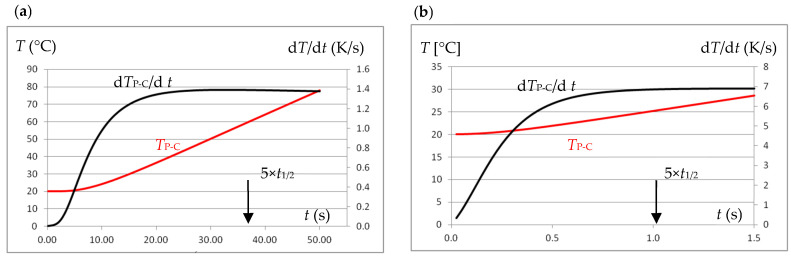
Temperature and its derivative waveforms at point P-C when excitation with a current of effective value *I* = 100 A and frequency *f* = 40 kHz: (**a**) for a sample made of 0H18N9 steel, (**b**) for a sample made of graphite.

Step excitation using the same effective excitation current causes varying temperature increases at the P-C control point of the tested sample, depending on the electrical and magnetic parameters. At the same time, depending on the thermal parameters, the maximum temperature derivative value is obtained at different times after the excitation occurs, as shown in Figure 3. It was established [11,12] that the duration of the step excitation equal to Δ*t*_step_ = 5·*t*_1/2_ is sufficient to achieve the maximum value of the temperature derivative, while the current intensity should be selected using the results of the initial test so that the temperature increase is at the level of several degrees.

The resistivity of the sample (charge) material influences the substitute values of resistance *R*_ch_ and reactance *X*_ch_ of the charge, as well as the measurable values of resistance *R*_IHS_ and reactance *X*_IHS_ of the inductor-charge system (induction heating system IHS). However, the changes in *R*_IHS_(*ρ*) and *X*_IHS_(*ρ*) that could be measured, even on the same test stand as the thermal parameters (Figure 1), are relatively small, and the nature of the changes may even be non-monotonic. In practice, this excludes the effective use of this method for indirect resistivity measurement of the sample material, even for non-magnetic materials. For this reason, the resistivity measurements were based on measuring changes in the magnetic flux *Φ*_in_ penetrating the charge (see the coil for measuring *Φ*_in_, Figure 1b). For practical reasons, the diameter of the measuring coil must be larger than the diameter of the charge, which weakens the effect of changes in the resistivity of the sample material on the measured value of the magnetic flux, which is determined (for a given f) by the value of the voltage *U*_coil_ induced in the measuring coil. As established in [12], the value of the induced voltage *U*_coil_ depends on the product of the resistivity and the relative magnetic permeability *ρ*·*μ*_r_. This allows the use of the *U*_coil_ voltage measurement to determine the resistivity of both non-magnetic and magnetic materials, provided that the value of their magnetic permeability is known. To improve measurement accuracy (especially for magnetic materials), it is recommended that the *U*_coil_ measurement be performed at the highest possible magnetic field strength (high exciter current). Therefore, the *U*_coil_ voltage measurement is performed simultaneously with the thermal diffusivity measurement, using a high current step excitation.

## 3. Magnetic Permeability Measurement Module

**Figure 4:**
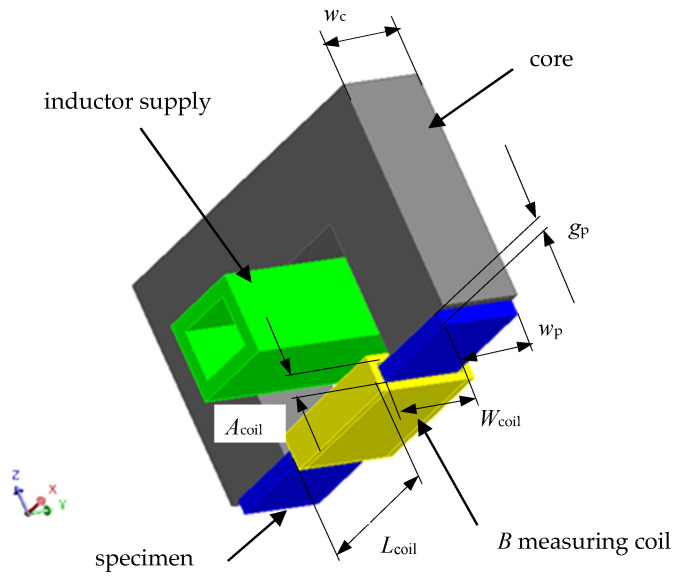
Schematic sketch of the measurement system for determining the magnetic properties of the tested material.

For conductive magnetic materials, determining thermal diffusivity, heat capacity, and resistivity using the procedures described above requires knowledge of the material’s magnetic properties. This requires information concerning the functional dependence of magnetic permeability (or the maximum magnetic induction *B*_m_) on the maximum magnetic field strength *μ*(*H*_m_), especially at relatively high magnetic field strengths (typical for induction heating). It is very difficult to determine these dependencies for the test sample in Figure 1b, especially at kilohertz frequencies. For this reason, it was decided to conduct magnetic property tests on a flat strip-shaped sample with a thickness of approximately 0.5 mm, a shape easily obtained under typical workshop conditions. Considering the skin effect (particularly strong for magnetic materials), this relatively large sample thickness leads to the need to reduce the frequency of the current driving signal to the low kilohertz range. The functional outline of the proposed measurement system is schematically presented in Figure 4.

**Figure 5:**
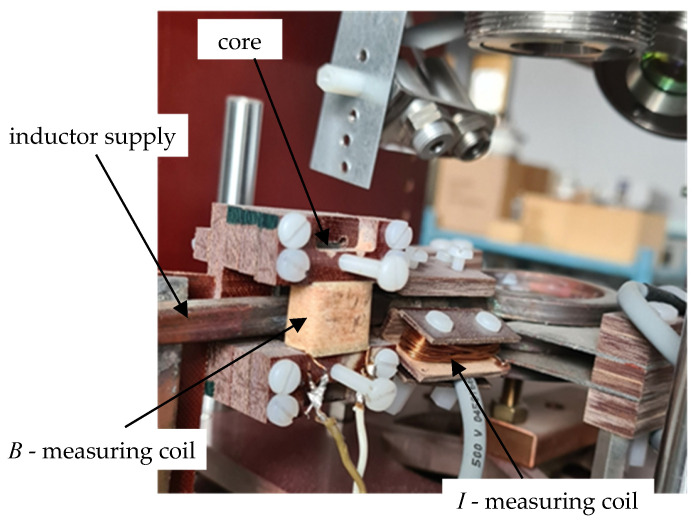
View of the measuring system for determining the magnetic properties of the tested material.

The measuring system for determining magnetic properties was built on the inductor supply line of the measuring station shown in Figure 1, which allowed for significant integration of this measurement with previous measurements of electrical and thermal parameters. A view of the added section of the measuring station is shown in Figure 5. Measurement signals from both the coil for indirect measurement of magnetic induction *B* and the coil for measuring the inductor current *I* extended the capabilities of the measuring block (Figure 1a) and were transmitted to the Host computer (Figure 1a).

It should again be emphasized that, in the case of induction heating, the ferromagnetic charge material is typically exposed to a magnetic field with a high maximum intensity. Therefore, the initial section (very low maximum values of *H*_m_) of the magnetization curve *B*_m_(*H*_m_) is not significant when determining the parameters of induction heating processes. This leads to the need for information concerning the magnetization curve (magnetic permeability), primarily for relatively high (at least thousands of A/m) magnetic field strengths. In the proposed measurement method, a ferrite material was used as the magnetic core, a material with high magnetic permeability but low saturation induction *J*_s_. To ensure that the magnetic voltage drop in the closed core-sample magnetic circuit (Figure 4) for the magnetic flux *Φ* induced by the inductor current occurs almost exclusively along the path of the tested sample, significant differences in the cross-sectional areas of the *S*_r_ core and the *S*_p_ sample must be maintained. This requires (apart from the skin effect) a relatively small sample thickness. A thickness of *g*_p_ = 0.5 mm was assumed as the starting point.

Assuming a sinusoidal current waveform of the inductor, the value of the amplitude *U*_m_ of the voltage induced in the measuring coil (covering the strictly tested sample) can be [15] expressed by the relationship (6), which makes it possible to calculate the maximum value of the magnetic induction *B*_m_:

(6) $$U_{m}=z\cdot \omega \cdot \Phi_{m}=z\cdot S_{p}\cdot \omega \cdot B_{m\;}\;\;\;\;\;\;\to \;\;\;\;\;\;B_{m}=\frac{U_{m}}{z\cdot \omega \cdot S_{p}}$$

In relation (6), it was assumed that the entire magnetic flux with amplitude *Φ*_m_ penetrating the measuring coil will be confined in the magnetic sample and will be evenly distributed in it.

**Figure 6:**
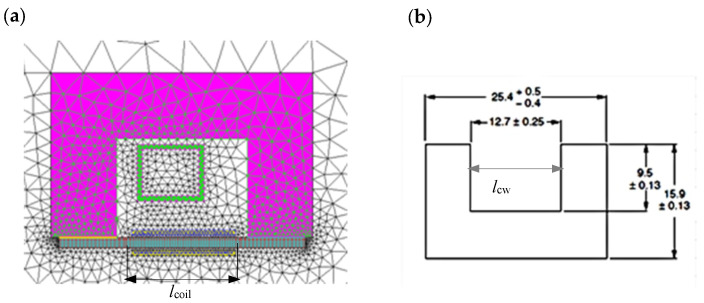
Model of the measurement system: (**a**) 2D simulation model; (**b**) core dimensions.

Using simulation calculations, we analyzed whether, and under what conditions, the proposed measurement system could be effectively used to determine the magnetic properties of the tested material. The initial analysis included a 2D model of the system shown in Figure 6. To reduce the impact of boundary phenomena at the core-to-sample magnetic flux transition, the length *l*_coil_ of the coil used to measure magnetic induction was assumed to be less than the linear dimension of the core window, assuming a value of *l*_coil_ = 11 mm.

Computational analysis using the finite element method (FEM) was performed using the commercial computer program Flux^®^, (Cedrat, Meylan, France) determining the influence of frequency, core material, and dimensions of the sample and measuring coil on the accuracy of determining the magnetic properties of the material of the tested sample.

It was assumed that the core (Figure 6b) is made of 3C90 material, the magnetization characteristics of which were approximated by the relationship (7), assuming *μ*_r,i_ = 2700, *J*_s_ = 0.49 T, and *a*_k_ = 0.1. Initially, SK10V steel was assumed as the material of the test sample, with the magnetization characteristics described by the relationship (6), with: *μ*_r,i_ = 489, *J*_s_ = 1.52 T, *a*_k_ = 0.873.

(7) $$\begin{array}{c} B_{m}\left(H_{m}\right)=\mu_{0}\cdot H_{m}+J_{s}\cdot \frac{H_{a}+1-\sqrt{{\left(H_{a}+1\right)}^{2}-4\cdot H_{a}\cdot \left(1-a_{k}\right)}}{2\cdot \left(1-a_{k}\right)}\\ H_{a}=\mu_{0}\cdot H_{m}\cdot \frac{\mu_{r,i}-1}{J_{s}} \end{array}$$

where: *a*_k_—“knee” coefficient, *μ*_r,i_—initial relative magnetic permeability, *J*_s_—saturation induction.

For samples with thicknesses close to *g*_p_ =0.5 mm, it was analyzed with what accuracy it is possible to assume that, in the analyzed core-sample system, the magnetic voltage drop occurs only along the length *l*_p_ close to the width *l*_cw_ =12.7 mm of the core window (Figure 6b) closed by the tested sample, and precisely along the length:

(8) $$l_{p}=l_{cw}+2\cdot g_{p}$$

With this assumption, the average value of the magnetic field strength amplitude *H*_m,p_ in the sample could be determined, using Ampere’s law [15], based on the amplitude *I*_m_ of the measured inductor current as:

(9) $$H_{m,p}=\frac{I_{m}}{l_{p}}$$

**Figure 7:**
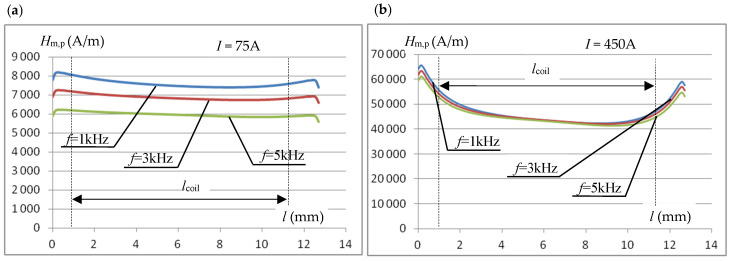
The effect of frequency on the distribution of the magnetic field intensity (across the core window width) in the middle of the sample thickness: (**a**) for the effective value of the inductor current *I* = 75 A; (**b**) for *I* = 450 A.

Figure 7 shows, for two values of the inductor current, the effect of frequency on the magnetic field strength distribution in the sample along a path equal to the core window width, halfway through the sample thickness.

The graphs show that the effect of frequency on the magnetic field strength distribution along the tested sample strongly depends on the inductor current. For a relatively low current with an effective value of *I* = 75 A (Figure 7a), a clear effect of frequency on the field strength is observed, but its distribution along the sample length is quite uniform. For much higher current values (*I* = 450 A) (and therefore magnetic field strength, Figure 7b), the effect of frequency is significantly smaller, but the distribution is more uneven, with a lower magnetic field value in the central part. The observed phenomena are, on the one hand, the result of the skin effect (which occurs more strongly at lower field strengths) in the sample, and, on the other hand, flux dispersion in the sample closing the magnetic circuit. At high magnetic field values (which is what we primarily encounter during induction heating), the sample enters the saturation region, which has a particularly strong impact on the magnetic flux distribution at the junction of the core and the measuring sample. This is also evidenced by the significantly increased difference in the field strength *H*_m,p_ at the right and left edges of the sample closing the core window (the inductor coil is placed asymmetrically in the core window, Figure 6). The assumed length of the measuring coil *l*_coil_, smaller than the width of the core window, Figure 6, helps to limit the impact of the phenomena described above on the value of the voltage induced in it.

**Figure 8:**
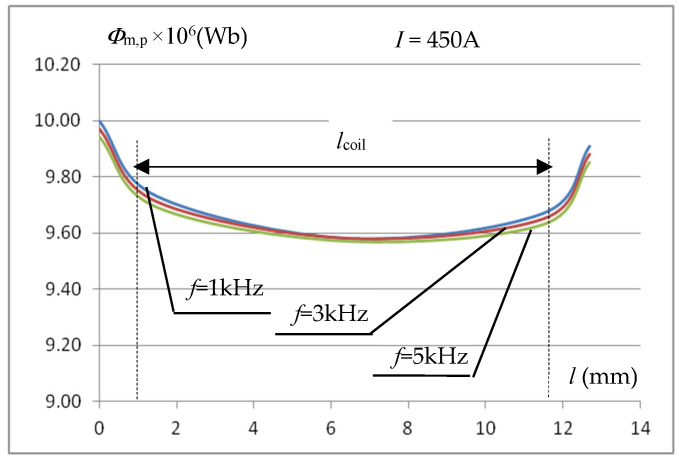
The influence of frequency on the magnetic flux distribution in the measuring sample across the width of the core window.

The value of the voltage induced in the measuring coil depends on the flux passing through it. This allows (6) to determine the average value of the magnetic induction in the sample volume covered by the measuring coil for the averaged value of the magnetic field strength in this area. The constancy of the magnetic flux along the length of the measuring coil improves the accuracy of the measurement of the *B*_m_(*H*_m_) characteristic. By integrating the distribution of the simulated magnetic induction value over the cross-section of the measuring sample (the variation of the magnetic field strength over the sample thickness is taken into account), the distribution of the magnetic flux *Φ*_m,p_ in the measuring sample over the width of the core window for the inductor current of 450 A was calculated and presented in Figure 8. The waveforms in Figure 8 indicate that the change in flux along the length of the measuring coil is small and amounts to approximately 1%.

**Figure 9:**
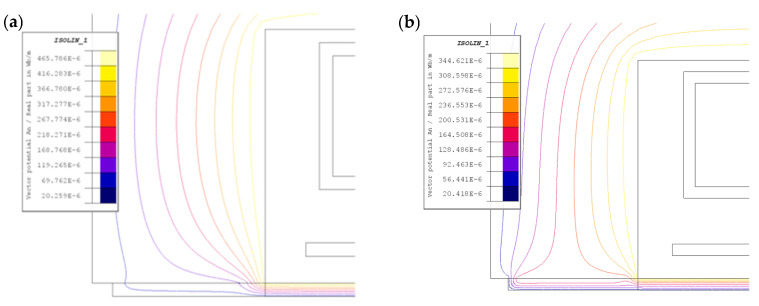
Distribution of magnetic field lines in the contact area between the core and the test samples at a small *I* = 25 A inductor current: (**a**) *f* = 1 kHz; (**b**) *f* = 5 kHz.

The accuracy of magnetic measurements in the setup shown in Figure 6 is not only influenced by the frequency and magnetic flux dispersion issues mentioned above. It is also influenced by the accuracy of the magnetic field strength estimation Formula (9), which assumes that the magnetic voltage drop in the magnetic core and in the part of the sample located under the core columns is neglected. This assumption can be considered justified only when there is a large difference in the reluctances of the core and the sample and when there is a strong curvature of the magnetic field lines in the core-sample contact area, Figure 9. However, these conditions do not necessarily have to be met, especially at low magnetic saturation of the sample material (low inductor current values) and higher frequencies.

Given the assumed dimensional proportions of the core and measuring sample cross-sections, the smaller “curvature” of the magnetic field lines in the core-sample region occurring with increasing frequency is particularly important, as shown in Figure 9b. This leads to an extension of the equivalent magnetic flux path in the sample, and therefore a decrease in the averaged value of the magnetic field intensity, as shown in Figure 7a; this is not taken into account by relation (9). In further considerations, at different frequencies and degrees of magnetic saturation of the sample, the magnitude of the resulting error was checked.

In the system considered for simulation, the calculated magnetic field intensity was integrated along the path *l*_p_ (in the middle of the sample thickness), determining its average value *H*_m,l_. Then, using the relationship (9), it was calculated what value of the inductor current *I*_1_ would give such an average value *H*_m,p_ = *H*_m,l_, i.e., the value resulting from the assumption that the entire magnetic voltage drop occurs only along the path of length *l*_p_. With the known, actual value of the inductor current *I* used in the simulation calculations, this makes it possible to assess the error *ε*_i_ (referred to the current intensity value) caused by the adopted assumption:

(10) $$\varepsilon_{i}=\frac{\left|I-I_{1}\right|}{I}\cdot 100\%$$

The error values *ε*_i_ for the sample currents at the considered frequencies *f* = 1 kHz, 3 kHz and 5 kHz are presented in Table 1.

The results of the error *ε*_i_ given in Table 1 show that, in the considered system, the adopted assumption of omitting the magnetic voltage drop in the core and the part of the sample under the core columns does not differ much from reality, for the case of a relatively low frequency (1 kHz) or high saturation of the sample material. In these cases, it can be assumed, in accordance with (9), that the entire magnetic voltage drop corresponding to the inductor current occurs in the sample along a path length *l*_p_ with a value defined by relationship (8) located at the center of the sample thickness. It should be noted that for an assumed sample thickness of *g* = 0.5 mm, the indicated significant errors appear at relatively low (as for induction heating applications) values of the magnetic field strength (lower magnetic field strength means higher magnetic permeability), and this is when the supply frequencies are significantly higher than *f* = 1 kHz. However, it is important to not overlook the uneven distribution of the magnetic field strength across the sample thickness *g*, caused mainly by the skin effect, which means that its value in the central part may not be representative of the entire cross-section. Therefore, the question arises as to how far the tested averaged magnetic field strength *H*_m,l_ deviates from the averaged field strength in the sample volume enclosed by the coil used to measure the averaged magnetic induction value, which is important from the point of view of the measured characteristic *B*_m_(*H*_m_) of the sample material. Using simulation results and integration over the sample cross-section area along the length enclosed by the coil, the averaged field strength *H*_m,c_ was calculated for the sample area inside the coil and compared with the *H*_m,p_ value from Equation (9), which allows for the calculation of the error *ε*_h_:

(11) $$\varepsilon_{h}=\frac{\left|H_{m,p}-H_{m,c}\right|}{H_{m,p}}\cdot 100\%$$

The error values *ε*_h_ for the exemplary values of the inductor current at the considered frequencies *f* = 1 kHz, 3 kHz and 5 kHz are presented in Table 2.

The error results from Table 2 confirm the conclusions resulting from the analysis of the error *ε*_i_ given in Table 1. They indicate that, for the assumed sample thickness *g* = 0.5 mm, the use of a current frequency of (1–2) kHz provides a high guarantee of a small (a few percent) error in determining the value of the magnetic field intensity in the sample (measuring coil area) using a simple relationship (8).

**Figure 10:**
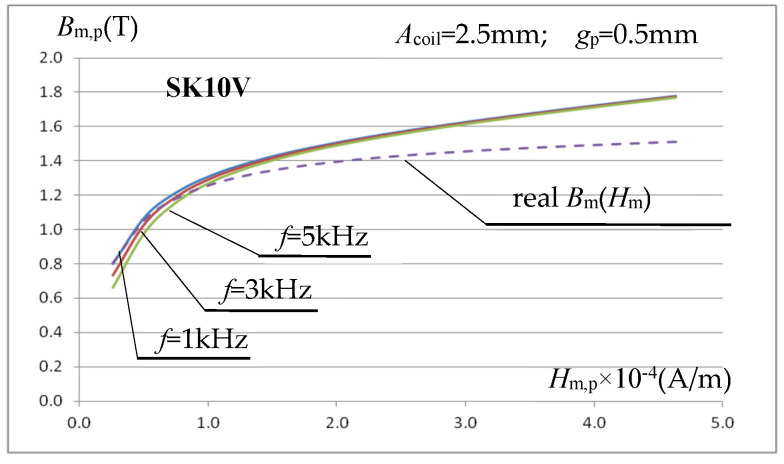
Magnetization characteristics for a sample made of SKV10 steel, with *B*_m,p_ determined from the relationship (6) and *H*_m,p_ determined from (9) using (8).

Using a 60-turn measuring coil (Figure 4 and Figure 6) with an internal width of *A*_coil_ = 2.5 mm (with a sample thickness of *g*_p_ = 0.5 mm, resulting in a gap of *ss* = 1 mm on each side of the sample), wound around the measuring sample, a simulation analysis was performed. This involved determining the magnetization characteristic *B*_m,p_(*H*_m,p_) of the sample material using relationships (6) and (9). The actual magnetization characteristic of the sample material is known in the simulation model, which makes it possible to assess the accuracy of the method for its determination using the described core-measuring coil system. Initially, SK10V steel was assumed as the sample material, with the magnetic parameters described in relationship (7) given above. Figure 10 shows, for three inductor current frequencies, the determined characteristic *B*_m,p_(*H*_m,p_) and the actual characteristic *B*_m_(*H*_m_) of the SK10V sample material assumed in the simulation model.

The determined characteristics *B*_m,p_(*H*_m,p_) presented in Figure 10 indicate that the method of determining the magnetic parameters of the sample material adopted in this work is a promising method, but with some significant drawbacks. At relatively low (for induction heating) values of the magnetic field strength calculated using relationship (8), the value of the inductance *B*_m,p_ calculated using relationship (6) is underestimated, and this error increases with increasing frequency. At high field strengths, close to saturation (important for induction heating applications), the value of the magnetic induction calculated based on the voltage induced in the measuring coil is clearly overestimated, regardless of the frequency of the driving signal.

**Figure 11:**
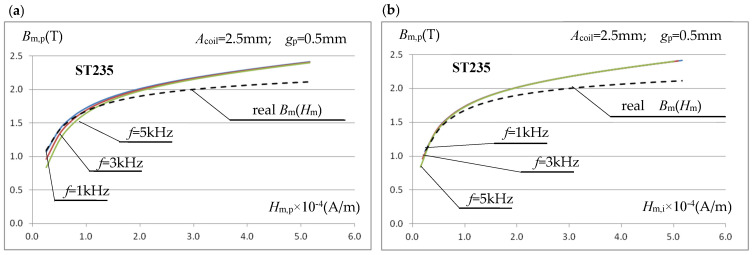
Magnetization characteristics for a sample made of ST235 steel, with *B*_m,p_ determined from the relationship (6) and: (**a**) *H*_m,p_ determined from (9) using (8); (**b**) *H*_m,i_ determined from the integration operation.

To determine the causes of the observed phenomenon, the simulation determination of the *B*_m,p_(*H*_m,p_) characteristics was repeated, but for a different test sample material with significantly different magnetization characteristics. The sample material was ST235 steel, characterized by a significantly higher saturation induction than SK10V. In the simulation calculations, the steel characteristics were described analytically by the relationship (7) with: *μ*_r,i_ =1100, *J*_S_ = 2.19 T, *a*_k_ = 2.30. Figure 11a shows the magnetization characteristics *B*_m,p_(*H*_m,p_) determined for ST235 (in the same way as before) for the frequency of the sinusoidal excitation signal of the inductor *f* = 1 kHz, 3 kHz and 5 kHz.

As expected, for a sample material with a stronger ferromagnetic effect, we observed an increase in the error in determining the magnetization characteristic *B*_m,p_(*H*_m,p_) for relatively small values of the magnetic field strength. This is mainly due to the error discussed above in determining the averaged field strength within the measurement sample using (9). To confirm this statement, Figure 11b presents the same magnetization characteristics for ST235, but the averaged values of the magnetic field strength *H*_m,i_ within the measurement sample were determined from the integration operation (and not from relation (9) as before).

**Figure 12:**
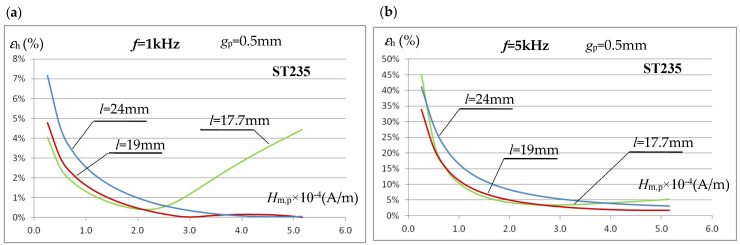
The influence of the sample length *l* on the error value *ε*_h_ of determining the averaged value of the magnetic field intensity by using the relationship (9): (**a**) for *f* = 1 kHz; (**b**) for *f* = 5 kHz.

The characteristics shown in Figure 11b, with correctly determined (from integration) values of the magnetic field strength *H*_m,i_, are almost identical for all tested frequencies and close to the real *B*_m_(*H*_m_) characteristic in the lower range of the tested field strengths. However, in a real, non-simulation measurement system, it is very difficult to determine the *H*_m,i_ value. We checked whether the accuracy of determining the averaged field strength value in the sample using Equation (9) could be improved by changing the length of the test sample (while maintaining the core dimensions). In the design solution analyzed so far, the test sample with a length of *l* = 24 mm almost completely enclosed the core (Figure 6). The magnetic voltage drop in the part of the sample in contact with the core strongly depends on both the frequency and the degree of sample saturation, as seen in Figure 9, which affects the error value *ε*_h_ (Table 2). For the ST235 material (where larger errors were observed than for SK10V steel), the effect of the sample length *l* on the error value *ε*_h_ of determining the magnetic field strength value using the relationship (9) was simulated. The analysis results for *l* = 24 mm, *l* = 19 mm (the sample overlaps approximately half the thickness of the core column), and *l* = 17.7 mm (the sample overlaps the core column by 2.5 mm) are shown in Figure 12a for *f* = 1 kHz and Figure 12b for *f* = 5 kHz.

The presented error characteristics for determining the averaged magnetic field strength using Equation (9) indicate that changing the relative sample length (relative to the magnetic core width) can slightly reduce the error. This applies primarily to the error for relatively small field strength values. The analyses performed indicate that to increase the accuracy of determining the averaged field strength using Equation (9) in the area of the sample covered by the measuring coil, the sample length can be selected to end at approximately half the core column thickness. While further shortening the sample length may reduce the error in some circumstances, it can also lead to an increase and should therefore not be used. It should be emphasized that the conclusion regarding shortening the sample length is justified only when its thickness is relatively small and oscillates around *g*_p_ = 0.5 mm, and the measurement frequencies used do not exceed several kilohertz.

**Figure 13:**
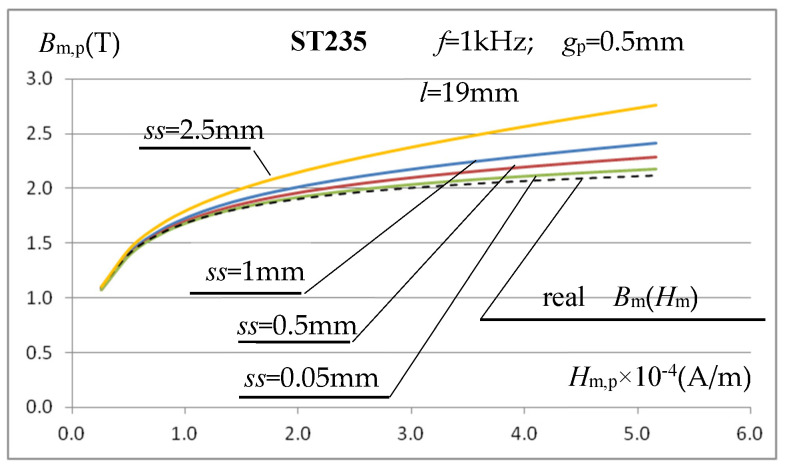
Influence of the coil-sample gap on the course of the calculated magnetization characteristic *B*_m,p_(*H*_m,p_).

The above analysis of the sample length selection indicates the possibility of some reduction in the error in determining the *B*_m_(*H*_m_) characteristic, but for relatively low field strength values, which are less important in induction heating applications. This does not solve the problem of large inaccuracies in determining the characteristic in areas close to the sample material saturation, as shown in Figure 11. The reason for this state of affairs can be traced to the increase in leakage flux around the test sample, whose magnetic permeability decreases in these field strength ranges. The air gap between the coil’s inner surface and the test sample assumed in simulation calculations so far was quite small, equal to *ss* = (*A*_coil_ − *g*_p_)/2 = 1 mm, and the thickness of the coil winding itself was only *g*_turn_ = 0.5 mm. Despite such relatively small dimensions, some of the flux inducing voltage *U*_m_ in the measuring coil is leakage flux confined to the coil-sample gap and the coil turns themselves. It should be noted that, with a sample thickness of *g*_p_ = 0.5 mm and high magnetic saturation, the value of this flux can have a noticeable, or even significant, effect on increasing the magnetic induction value calculated using relationship (6). The lower the magnetic permeability (higher saturation) of the sample material, the greater this effect will be. To verify the above hypothesis, simulation calculations were repeated for smaller gap values than before (*ss* = 1 mm), i.e., *ss* = 0.5 mm and *ss* = 0.05 mm, and for a very large gap of *ss* = 2.5 mm. The obtained *B*_m,p_(*H*_m,p_) characteristics for *f* = 1 kHz are shown in Figure 13.

The graphs in Figure 13 confirm the hypothesis regarding the need to minimize the coil-sample gap, particularly when measuring high magnetic field strengths, where the tested material is close to magnetic saturation. In practice, however, it is difficult to meet this requirement, as it is necessary to freely insert the tested samples (which, in practice, may vary slightly in thickness). Therefore, it was assumed that the gap cannot be smaller than *ss* = 0.5 mm. In such a case, the total air gap (without taking into account the winding thickness) in the measuring coil is still approximately twice the sample thickness. At magnetic field strengths exceeding 50 kA/m, typical for induction heating, this will still lead to significant errors, significantly exceeding 10% (Figure 13), in determining magnetic induction based on the voltage induced in the measuring coil and the relationship (6). Taking this into account, we decided to introduce a correction to Formula (6), taking into account the differences in the cross-sectional area of the sample *S*_p_ and the actual field for the magnetic flux flow inside the coil, which also includes a certain additional part of the cross-sectional area of her interior *S*_coil_ (i.e., the coil-sample air gap, where the gap assumed in the calculations in the direction perpendicular to the core column cannot protrude beyond the core by more than 10% of the core thickness) and the cross-sectional area *S*_turn_ of the winding layer up to half of its thickness. It was assumed that the voltage induced in the measuring coil is the result of three magnetic fluxes penetrating it: *Φ*_m,p_ in the sample, *Φ*_m,p_coil_ in the air gap, and *Φ*_m,p_turn_ in the coil windings, which, assuming their constant distribution in individual areas, leads to the following relationship:

(12) $$\begin{array}{cc} U_{m}=z\cdot \omega \cdot \Phi_{m} & =z\cdot \omega \cdot \left(\Phi_{m,p}+\Phi_{m,p_{\_coil}}+\Phi_{m,turn}\right)=z\cdot \omega \cdot \left[B_{m,p}\cdot S_{p}+\frac{B_{m,p}}{\mu_{r,p}}\cdot \left(S_{coil}-S_{p}+S_{turn}\right)\right]\\ & =z\cdot \omega \cdot B_{m,p}\cdot \left(S_{p}+\frac{S_{turn}+S_{coil}-S_{p}}{\mu_{r,p}}\right)=z\cdot \omega \cdot B_{m,p}\cdot \frac{S_{p}\cdot \left(\mu_{r,p}-1\right)+S_{turn}+S_{coil}}{\mu_{r,p}} \end{array}$$

where: *μ*_r,p_—relative magnetic permeability of the sample material.

Taking into account (12), the dependence on the magnetic induction in the sample will take the form:

(13) $$B_{m,p}=\frac{U_{m}\cdot \mu_{r,p}}{z\cdot \omega \cdot [S_{p}\cdot \left(\mu_{r,p}-1\right)+S_{turn}+S_{coil}]}$$

Using Equation (13) requires knowledge of the relative magnetic permeability of the sample material *μ*_r,p_ for the magnetic field strength corresponding to the calculated induction, i.e., it requires prior information on the value of the quantity being calculated. As a first approximation, we can assume the value of the magnetic induction calculated using Equation (6), which allows us to determine the relative permeability *μ*_r,p_ used in (13). The resulting magnetic induction value is then used in an iterative cycle to determine a new magnetic permeability value until its relative change falls below the assumed tolerance. Two or three iterative cycles typically achieve an accuracy of 1–2%.

**Figure 14:**
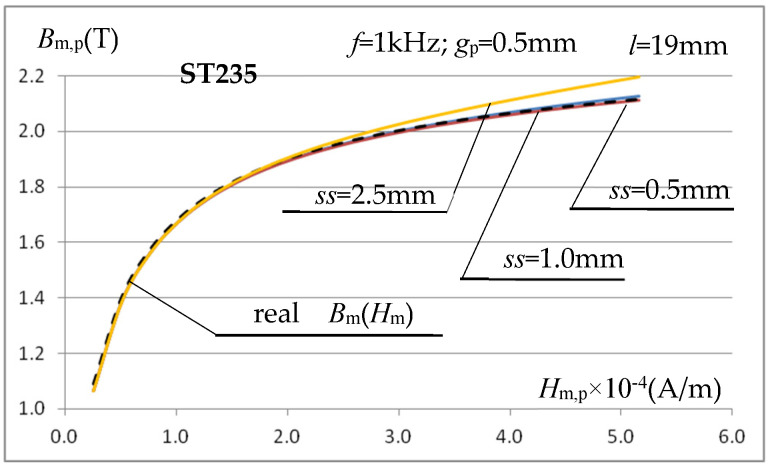
Magnetization characteristics of ST235 steel at frequency *f* = 1 kHz and gap *ss* = 0.5 mm, *ss* = 1 mm and *ss* = 2.5 mm calculated using relations (13) and (9).

Using (13), the magnetization characteristics *B*_m,p_(*H*_m,p_) for gaps *ss* = 2.5 mm, *ss* = 1 mm, and *ss* = 0.5 mm at *f* = 1 kHz were determined again for the ST235 steel and presented in Figure 14.

**Figure 15:**
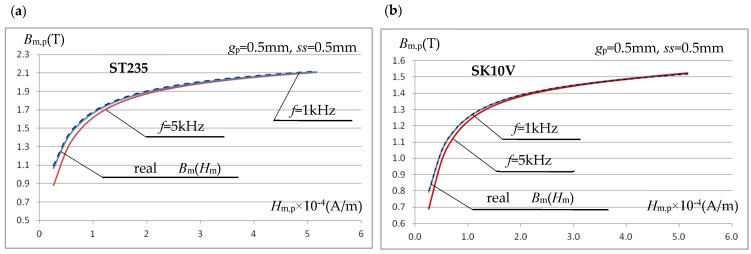
The effect of frequency on the accuracy of calculating the magnetization characteristics using relations (13) and (9): (**a**) steel ST235; (**b**) steel SK10V.

The presented characteristics show that when using relationship (13), even at magnetic field strength values close to saturation, the calculated value of magnetic induction *B*_m,p_ depends very little (compare Figure 13) on the value of the gap *ss* between the inner surface of the measuring coil and the tested sample. At high magnetic field strengths, the calculated value of *B*_m,p_ is very close (at *ss* = 0.5 mm and *f* = 1 kHz with an error below 1%) to the value resulting from the magnetization characteristics assumed in the simulation. Only for relatively very large (relative to the sample thickness) gaps do errors of several percent occur. As the inductor current frequency increases, the error in calculating *B*_m,p_ increases slightly, as shown in Figure 15a, but this mainly concerns relatively low values of the magnetic field strength, as shown for the frequency *f* = 5 kHz.

In the considered measurement system, the errors in determining the *B*_m,p_(*H*_m,p_) characteristic, using the relations (9) and (13), decrease with the decrease in saturation induction and initial magnetic permeability of the tested sample material, as shown in Figure 15b for SK10V steel (characterized by significantly lower saturation induction and initial permeability than ST235).

The results of the 2D simulation considerations conducted above indicate that, for samples approximately 0.5 mm thick and with a length enclosing the U-type core at approximately half the core column thickness, it is possible (in the arrangement shown in Figure 6) to determine the magnetization characteristics *B*_m,p_(*H*_m,p_) of the tested sample material with an error not exceeding a few percent. For this purpose, Equation (9), using Equation (8), should be used to determine the average field strength value, and Equation (13) should be used to determine the magnetic induction. This applies to relatively large values of magnetic field strength, typical for induction heating, exceeding 5 kA/m (which translates into surface densities of power penetrating the charge of the order of several to several dozen W/cm^2^).

**Figure 16:**
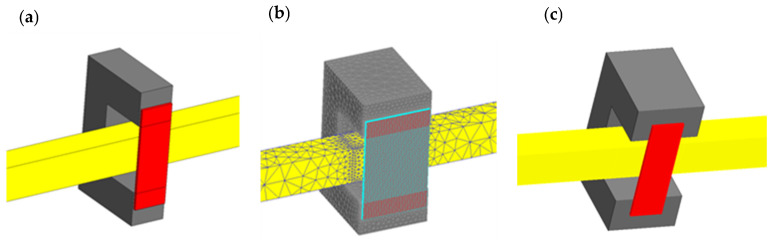
Considered 3D models: (**a**) one core and a sample with a width of *w*_p_ = 6 mm, (**b**) two cores and a sample of *w*_p_ = 12 mm, (**c**) two cores and a sample of *w*_p_ = 6 mm.

The simulations were conducted in a 2D system (Figure 6), which did not take into account changes in the field distribution in the direction perpendicular to the end face of the U-type magnetic core, assuming that the system is infinitely long in this direction. In reality, the cross-sections of a column of U-type cores usually do not deviate significantly from the square; in the analyzed case, this leads to a single core dimension of *w*_c_ = 6.4 mm (Figure 4). Taking into account practical considerations, it was assumed that the sample width should be relatively small, and the sample width was therefore assumed to be *w*_p_ = 6 mm (Figure 4). With such dimensional proportions, the analyzed 2D model of the core-sample system may introduce significant computational simplifications and requires verification. Based on the assumed geometric dimensions, simulation verification was performed for three 3D models:

- model containing a single U-shaped core (*w*_c_ = 6.4 mm) and a sample with a width of *w*_p_ = 6 mm, Figure 16a;
- model containing two U-shaped cores (*w*_c_ = 2 × 6.4 = 12.8 mm) and a sample with a width of *w*_p_ = 2 × 6 = 12 mm, Figure 16b;
- model containing two U-shaped cores (*w*_c_ = 2 × 6.4 = 12.8 mm) and a centrally placed sample with a width of *w*_p_ = 6 mm, Figure 16c.

**Figure 17:**
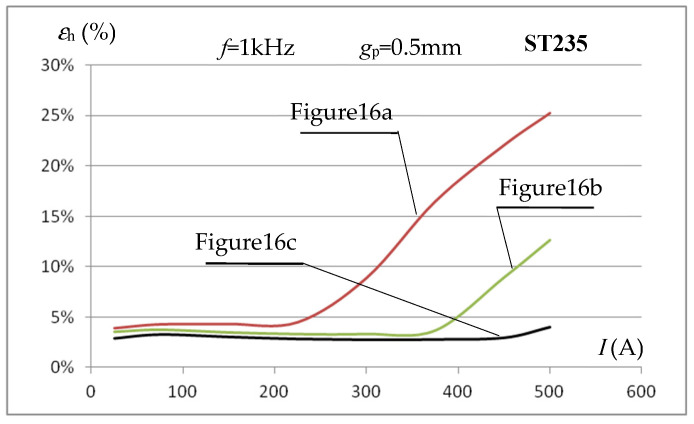
The influence of the dimensional proportions of the test sample width and the core on the accuracy of determining the average value of the magnetic field intensity in the sample using the relationship (9) in 3D calculations.

For the considered systems, using the obtained simulation results and integration over the sample volume along the core window length, the averaged value of the field strength *H*_m,c_ was calculated for the tested sample area. Using (11), this makes it possibleto evaluate the error *ε*_h_ in determining the average value of the magnetic field strength *H*_m,p_ using the relationship (9). Figure 17 shows the dependence of the error *ε*_h_ in determining the average value of the magnetic field strength for various values of the inductor current for a sample made of ST235 steel and a frequency of *f* = 1 kHz and the three considered dimensional proportions (Figure 16).

The graphs in Figure 17 show that, both when using one and two cores (and a sample with a width *w*_p_ similar to the core dimensions), the error values for determining the average magnetic field strength in the tested sample using relationship (9) differ significantly from the results from the 2D model. This applies especially to higher current values (magnetic field strength) when we are dealing with higher saturation of the sample material.

The error results obtained for the case from Figure 16c, i.e., when the sample width is significantly smaller than the core width, are very similar to those obtained in the 2D model; this applies to both small and large values of the magnetic field strength (inductor current values). The verification performed indicates that the results of the 2D analysis performed above can be fully reliable, provided they concern a system similar to the one shown in Figure 16c. This leads to the conclusion that, when determining the magnetization characteristics *B*_m_(*H*_m_) on the measuring stand, as shown in Figure 5, the field strength value can be determined by measuring the current intensity in the inductor and using the relationship (9), but the width of the measuring sample must be significantly smaller than the total width of the cores.

**Figure 18:**
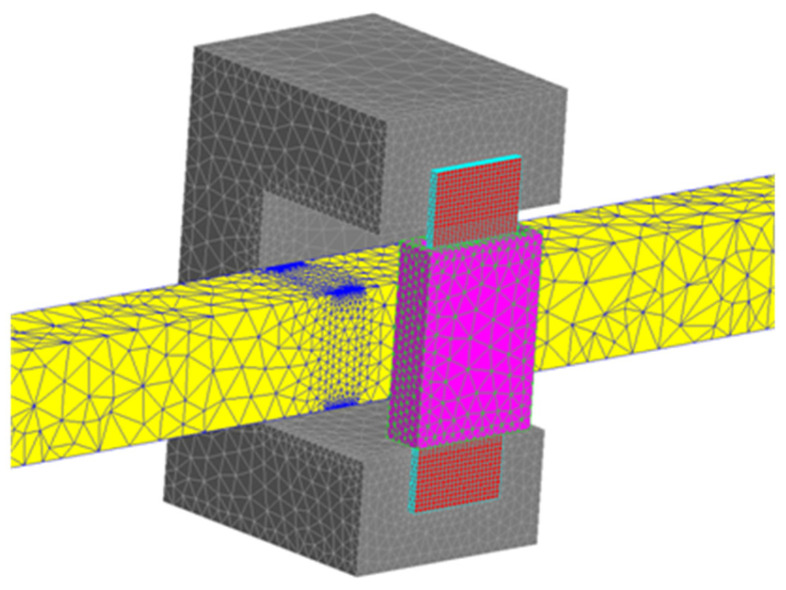
3D simulation model of the considered system for measuring the magnetization characteristics *B*_m_(*H*_m_).

Based on the conclusions from the analysis carried out above, a simulation verification was carried out for the 3D system from Figure 16c, but equipped with a measuring coil, as shown in Figure 18. As in the case of the considerations for the 2D model, the voltage induced in the measuring coil is the basis for determining the value of the averaged magnetic induction *B*_m_.

**Figure 19:**
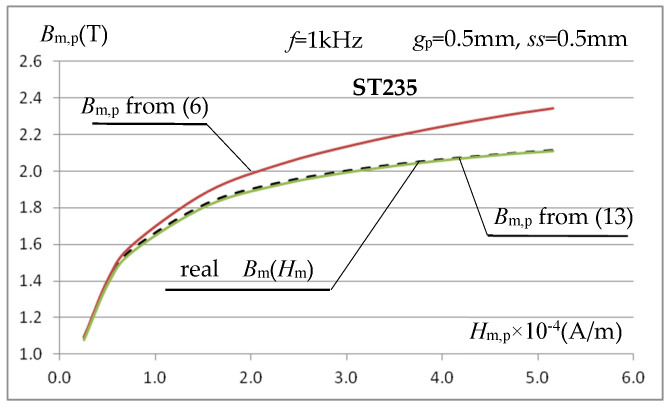
The influence of the method of calculating magnetic induction in the 3D model on the course of the determined magnetization characteristics.

3D simulation verification calculations were performed (similarly to the 2D model) for a sample made of ST235 steel, with a sample-coil air gap of *ss* = 0.5 mm and an inductor current frequency of *f* = 1 kHz. Figure 19 presents the magnetization characteristics *B*_m,p_(*H*_m,p_) determined based on the voltage induced in the measuring coil. For the effective value of the inductor current varying in the range *I* = (25–500) A, the average value of the magnetic field strength was determined based on Equation (9), while the magnetic induction value was determined from the induced voltage using Equation (6), as well as taking into account the effect of the coil-sample gap, i.e., using Equation (13).

The 3D results presented in Figure 19 confirmed a high agreement between the magnetization characteristics *B*_m,p_(*H*_m,p_) obtained using relations (9) and (13) and the actual characteristics *B*_m_(*H*_m_) of the sample material. For the system shown in Figure 16c (the tested sample was clearly narrower than the core), this confirmed the conclusions from the 2D analysis, which justifies its use for further analysis of this type of measurement system.

The 2D and 3D simulation calculations presented above were conducted as AC calculations, with inductor current frequencies ranging from 1 to 5 kHz. For the magnetically nonlinear system under consideration, this involves obvious simplifications, assuming sinusoidal magnetic induction and field strength waveforms. However, in the case of the analyzed measurement station (Figure 1), which is designed to enable the measurement of temperature characteristics of many material parameters, we are faced with a more significant physical problem resulting from technical limitations. This stems from the desire to use a single inverter-based, resonant power source in the measurement station to measure both thermal diffusivity, heat capacity, and resistivity, as well as, in the case of magnetic materials, the magnetization characteristics. For the first group of measurements, frequencies of the order of (30–50) kHz are appropriate [11,12], while for determining the magnetization characteristics, a maximum of several kilohertz is used. In the case of a resonant inverter, such a significant frequency reduction would lead to operational problems significantly hindering the operation of the station, and the use of PWM technology for measuring the magnetization characteristics is not possible due to harmonics. Taking into account the above operational considerations, the possibility of using a triangular signal to measure the magnetization characteristics *B*_m,p_(*H*_m,p_) was considered in the analyzed measurement station. Such a change in the measurement signal can be relatively easily implemented by the station’s control systems.

The possibility of effectively using the triangular signal in the considered measurement system was verified in the 2D simulation system presented above. Using the analysis of the transient electromagnetic field, the dependence of the instantaneous value *u* of the voltage induced in the *z*-turn measuring coil on the inductor current having a triangular waveform with period *T*_T_ was investigated. According to the law of electromagnetic induction, this leads to a relationship that allows for the calculation of the maximum value *Φ*_m_ of the flux penetrating the measuring coil:

(14) $$u=z\cdot \frac{d\Phi }{dt}\;\;\to \;\;\int u\cdot dt=z\;\cdot \Phi \to \Phi_{m}=\frac{1}{z}\underset{\tau \in [0,T_{T}]}{\max}(\int_{0}^{\tau }u\cdot dt)$$

The maximum value of the magnetic field intensity *H*_m,p_ in the sample can, as before, be calculated from the relation (9) using relation (8). The flux inducing the voltage in the coil penetrates the entire interior of the coil, not just the test sample, which (assuming uniform flux distribution in the individual areas), similarly to (13), leads to the relation:

(15) $$B_{m,p}=\frac{\underset{\tau \in [0,T_{T}]}{\max}(\int_{0}^{\tau }u\cdot dt)\cdot \mu_{r,p}}{z\cdot [S_{p}\cdot \left(\mu_{r,p}-1\right)+S_{turn}+S_{coil}]}$$

**Figure 20:**
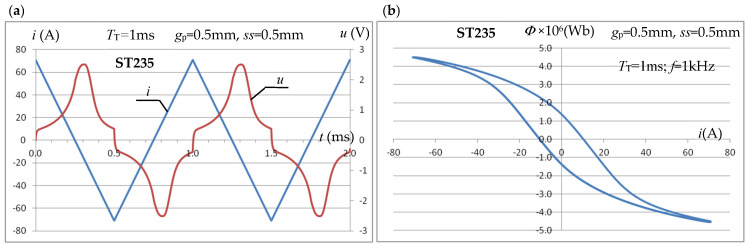
Waveforms of i(t) of the excitation current of the inductor and u(t) of the induced voltage (**a**) and *Φ* of the magnetic flux (**b**) in the measuring coil for the triangular shape of the inductor current with the maximum value *I*_m_ = 70.7 A.

Figure 20 shows exemplary time courses of the instantaneous value *i*(*t*) of the inductor current, the voltage *u*(*t*) induced in the measuring coil, and the magnetic flux *Φ*(*i*) in the coil as a function of the inductor current for a sample made of ST235 steel and a triangular current of inductor with a maximum value of *I*_m_ = 70.7 A and a period of *T*_T_ = 1 ms (frequency *f* = 1 kHz).

**Figure 21:**
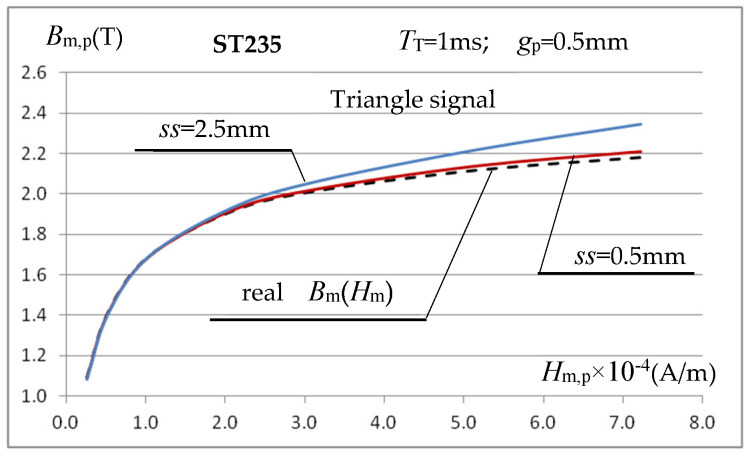
Magnetization characteristics of ST235 steel determined by a triangular signal with a period *T*_T_ = 1 ms at a gap of *ss* = 0.5 mm and *ss* = 2.5 mm using relations (15) and (9).

The conducted tests allow us to conclude that the use of a triangular signal in the considered measurement system is fully justified. Technically, it is easier to obtain in the considered measurement setup and produces very similar results to a sinusoidal signal. Figure 21 shows an example of the characteristics *B*_m,p_(*H*_m,p_) for ST235 steel, obtained for a triangular signal with a period *T*_T_ = 1 ms and a sample with a thickness of *g*_p_ = 0.5 mm.

The waveforms in Figure 21 indicate that, for a small air gap *ss* between the coil and the sample, the determined magnetization characteristic is very close to the actual characteristics of the sample material. If the gap width is significantly larger than the sample thickness *ss* = 2.5 mm (*g*_p_ = 0.5 mm), an error of several percent in the determination of *B*_m,p_ is observed (similarly to the sinusoidal signal), which increases with the increase in the magnetic field strength; such situations should be avoided.

## 4. Procedure for Measuring Thermal, Electrical, and Magnetic Properties of Ferromagnetic Materials

The test stand shown in Figure 1 allows for the determination of temperature characteristics of heat capacity *χ*(*T*) = *c*(*T*)·*ρ*_m_ and thermal diffusivity *a*(*T*) = *k*(*T*)/*χ*(*T*), as well as the resistivity *ρ*(*T*) of conductive material samples in a single measurement cycle. As described above, research is currently underway to expand the test stand’s capabilities with an integrated module for determining the magnetization characteristics *B*(*H*).

**Figure 22:**
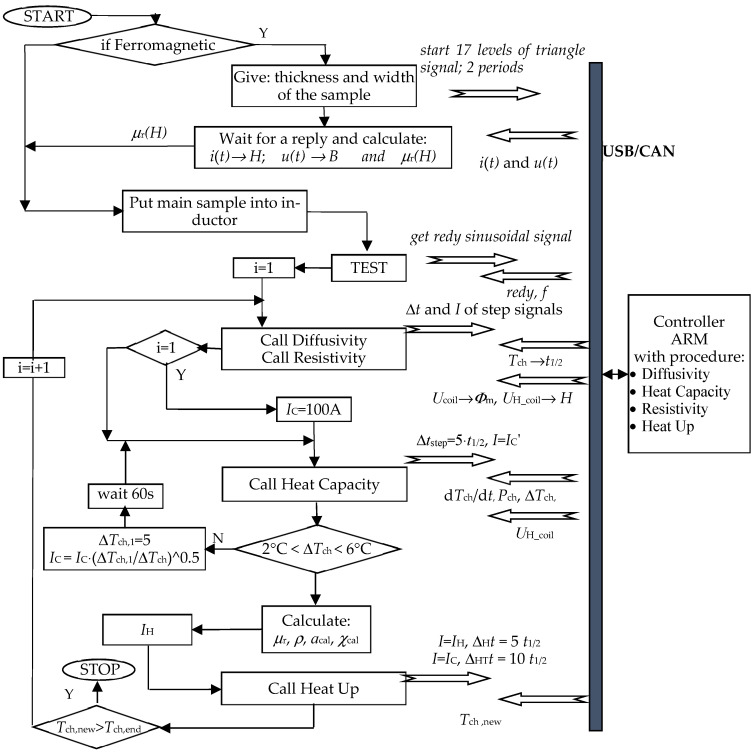
Simplified algorithm of the stand for estimating the magnetization characteristics and temperature characteristics of thermal diffusivity, heat capacity, and resistivity of conductive materials. Where *I*_H_ calculated as in [12].

A simplified algorithm of the test stand operation, including the module for determining the magnetization characteristics, is shown in Figure 22.

In the case of ferromagnetic materials, information concerning magnetic properties, defined by the relationship between relative magnetic permeability *μ*_r_(*H*) and magnetic field strength, is also necessary to determine the temperature characteristics of resistivity. Therefore, the measurement procedure for determining the magnetization characteristics at the station shown in Figure 1 was planned to precede the measurement cycle for determining the thermal-electric temperature characteristics. Magnetic measurements are performed at the same measurement station using the same power source and computer control and measurement system, but they are based on a different test sample (different shape) and use a triangular current signal, not a sinusoidal one, and at a significantly lower frequency, which (this has not been investigated) may reduce measurement accuracy.

At the initial stage of experimental verification, the correctness of determining the magnetization characteristic *B*(*H*) was initially checked in the context of the conclusions presented above, based on computer simulations. The tests were performed primarily on a sample made of ST235 steel, as well as SK10V steel with known magnetization characteristics placed in a measuring coil with *z*= 60 turns (Figure 5) and a significantly larger cross-section than the sample. The width of the test sample was similar to the width of the magnetic core, which corresponded to the variant in Figure 16a. The measurements were performed for 17 levels of the triangular inductor current signal with frequencies in the range *f* = (1.5–5) kHz.

Detailed dimensions (Figure 4) of the test setup in Figure 5:

- test sample—*g*_p_ = 0.55 mm, *w*_p_ = 6 mm;
- measuring coil—*A*_coil_ = 5.5 mm, *W*_coil_ = 12.2 mm, *z* = 60;
- core—*w*_c_ = 6.4 mm.

**Figure 23:**
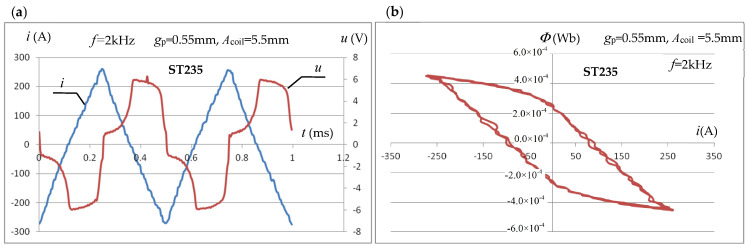
The measured waveforms of *i*(*t*) of the forcing current and *u*(*t*) of the induced voltage (**a**) and *Φ*(*i*) of the magnetic flux (**b**) in the measuring coil at the maximum current value *I*_m_ = 270 A.

Figure 23a shows, for example, for the amplitude of the triangular forcing current *I*_m_ = 270 A and *f* = 2 kHz, the waveforms of the instantaneous values *i*(*t*) of the inductor forcing current and the voltage *u*(*t*) induced in the measuring coil subjected to median filtering. Figure 23b shows the waveform of the magnetic flux *Φ*(*i*) in the coil obtained from integration as a function of the instantaneous values of the inductor current.

**Figure 24:**
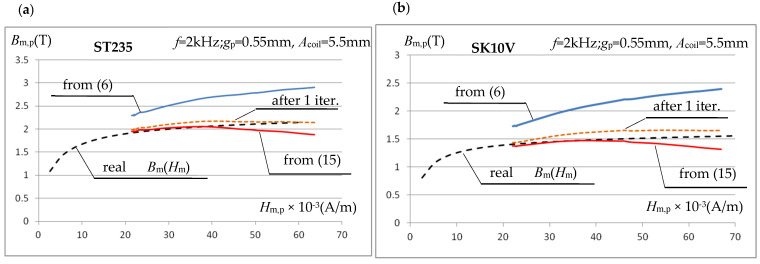
Magnetization characteristics of (**a**) ST235 and (**b**) SK10V steel obtained in the tested measuring system. Triangular signals *f* = 2 kHz.

For the 17 levels of maximum current values of the triangular waveform measured, the corresponding maximum values of the magnetic field strength were determined from Equations (7) and (8). For each of them, the maximum value of the magnetic induction was determined based on the integral of the measured instantaneous voltage values induced in the measuring coil. Initially, Equation (6) was used for the calculations, followed by Equation (15) in an iterative cycle. Figure 24 presents the magnetization characteristics *B*_m,p_(*H*_m,p_) obtained in the tested measurement system for ST235 and SK10V steels, using calculations based on (6), as well as for the first and last (error below 1%) iteration cycle using (15).

The final course of the magnetization characteristic *B*_m,p_(*H*_m,p_) obtained from experimental measurements largely confirms the conclusions presented above, resulting from simulation calculations. At relatively high values of the magnetic field strength (of the order of 30 kA/m), corrections to the inductance value, performed using Equation (15), significantly reduce the measurement error, even (as in the presented example) at a very large value of the sample-to-test coil gap *ss*. The presented example also suggests that, at magnetic field strengths exceeding 40 kA/m, the correction leads to a significantly excessive reduction in the magnetic induction value, and a decrease in the induction *B*_m,p_ is observed with increasing magnetic field strength *H*_m,p_. This is an erroneous suggestion. The observed phenomenon results from an erroneous course of the initial characteristic *B*_m,p_(*H*_m,p_); see the blue curve in Figure 24. Above a value of approximately 40 kA/m, a clear decrease in the rate of increase in induction *B*_m,p_ is observed, which results from errors (too large values) in determining the magnetic field strength using Equations (8) and (9). As shown above, Figure 17, when the sample width *w*_p_ is close to the core width *w*_c_ (variant in Figure 16a), the use of the above equations to determine the magnetic field strength leads to a drastic increase in error at current intensities that cause significant magnetic saturation of the tested sample. The experimental studies confirmed the validity of the previously formulated conclusion.

## 5. Conclusions

The correct course of technological processes, including heating processes, usually depends on the material parameters of the workpieces. Accurate knowledge of these parameters often requires their measurement, as literature values can be difficult to obtain or imprecise. In the case of induction heating systems, where thermal energy is generated directly in the heated object (load) using electromagnetic phenomena, this information is particularly important. It concerns, at least, the thermal and electrical-magnetic parameters. Although these parameters jointly influence the induction heating process, their experimental determination is usually quite complex, requiring completely different instruments and measurement methods, which translates into significant costs. This paper addresses this problem through the concept of a measurement station enabling the experimental estimation, with the highest possible accuracy, of thermal parameters (heat capacity and diffusivity), electrical parameters (resistivity), and magnetic parameters (magnetic permeability). In the case of thermal-electrical parameters, this involves the simultaneous determination of the temperature characteristics of these properties (in a single automatic measurement cycle). In the case of ferromagnetic materials, the correct determination of these characteristics requires information concerning their magnetization characteristics. This paper focuses on this aspect, examining the feasibility of determining magnetization characteristics using the same test stand with its power supply, control systems, and software, but in a separate test cycle using a test sample of a different shape. Simulation studies allowed us both to develop a method for achieving this goal and to investigate the influence of the shape and relative proportions of the measurement system components on the accuracy of magnetization characteristics measurement. The analyses indicate that the test stand can effectively determine magnetization characteristics for flat samples less than 1 mm thick using triangular current signals with frequencies of 1–2 kHz. The test magnetic circuit can be based on a U-type ferrite core, in which the test sample encloses the core, overlapping at least half of the core column. The width of the core used (Figure 16) should be at least twice the width *w*_p_ of the sample. In such cases, the average magnetic field strength of the sample can be determined based on relations (8) and (9). It is recommended that the sample-to-researcher coil gap be as small as possible. Correction for the effect of the technically necessary gap on the accuracy of determining the magnetic induction in the sample can be based on the use of relationship (15).

The above requirements enable the discussed measurement system to estimate the magnetic permeability characteristics with an error of several to a dozen or so percent in the range of relatively large (typical for induction heating) values of magnetic field strength, i.e., above a dozen or so kA/m.

## Figures and Tables

**Table 1:** Error from assuming the magnetic voltage drop only on the *l*_p_ path in the middle part of the sample thickness.

	*I* = 25 A		*I* = 300 A		*I* = 450 A	
*f* (kHz)	*I*_1_ (A)	*ε*_i_ (%)	*I*_1_ (A)	*ε*_i_ (%)	*I*_1_ (A)	*ε*_i_ (%)
1	23.4	6	294.7	2	443.0	2
3	19.9	20	289.5	4	437.5	3
5	16.8	33	283.5	6	430.9	4

**Table 2:** The error in the calculation from (8) of the average value of the magnetic field strength in the sample area inside the measuring coil.

	*I* = 25 A	*I* = 75 A	*I* = 300 A	*I* = 450 A
*f* (kHz)	*ε*_h_ (%)	*ε*_h_ (%)	*ε*_h_ (%)	*ε*_h_ (%)
1	0.1	2.0	3.1	3.8
3	18.0	8.0	4.3	4.3
5	30.1	14.9	5.8	5.5

## Data Availability

The original contributions presented in this study are included in the article. Further inquiries can be directed to the corresponding author.
